# Can Aging in Place Be Cost Effective? A Systematic Review

**DOI:** 10.1371/journal.pone.0102705

**Published:** 2014-07-24

**Authors:** Erin M. Graybill, Peter McMeekin, John Wildman

**Affiliations:** 1 Institute of Health & Society, Newcastle University, Newcastle upon Tyne, United Kingdom; 2 Business School, Newcastle University, Newcastle upon Tyne, United Kingdom; University of Glasgow, United Kingdom

## Abstract

**Purpose of the Study:**

To systematically review cost, cost-minimization and cost-effectiveness studies for assisted living technologies (ALTs) that specifically enable older people to ‘age in place’ and highlight what further research is needed to inform decisions regarding aging in place.

**Design:**

People aged 65+ and their live-in carers (where applicable), using an ALT to age in place at home opposed to a community-dwelling arrangement.

**Methods:**

Studies were identified using a predefined search strategy on two key economic and cost evaluation databases NHS EED, HEED. Studies were assessed using methods recommended by the Campbell and Cochrane Economic Methods Group and presented in a narrative synthesis style.

**Results:**

Eight eligible studies were identified from North America spread over a diverse geographical range. The majority of studies reported the ALT intervention group as having lower resource use costs than the control group; though the low methodological quality and heterogeneity of the individual costs and outcomes reported across studies must be considered.

**Implications:**

The studies suggest that in some cases ALTs may reduce costs, though little data were identified and what there were was of poor quality. Methods to capture quality of life gains were not used, therefore potential effects on health and wellbeing may be missed. Further research is required using newer developments such as the capabilities approach. High quality studies assessing the cost-effectiveness of ALTs for ageing in place are required before robust conclusion on their use can be drawn.

## Introduction

Many adults want to grow old in their own homes (age in place) as opposed to a community living arrangement [1] [2], but there are challenges to independent living for healthy older adults as well as those who are unwell [3]. Aging populations require innovative solutions to the problems of maintaining independence, dignity and home care, and assisted living technologies (ALTs) may play a key role. ALTs can theoretically facilitate older people (those aged age 65+ [4]) aging in place and can be categorized into two groups. Firstly, *Home and Environmental Modifications*, which are technologies or modifications that can be installed or used within a home to promote independent living, usually by increasing mobility (e.g. ramp, adapted kitchen tools), or mediating risk of injury (e.g. grab rails in bath, wheelchair lift on stairs). Secondly, *Telemedicine* which includes any technology that provides remote communication between people in their home and healthcare or security professionals [5], [6] (e.g. home surveillance technology to monitor the condition of the older person by transmitting routine physiological data).

Although technological advancements continue to evolve, the healthcare industry has been hesitant to adapt [7]. ALT solutions need to be focussed on both improving the aging in place experience and helping to contain costs for individuals and the public purse. This paper aims to systematically review cost and cost-effectiveness studies for ALTs specifically for older people, and to highlight where research is needed in order to make a case for ALTs.

Health interventions are increasingly subjected to economic evaluation that combines information on effectiveness and cost to consider the cost-effectiveness of their implementation. There is a growing body of research evidence describing the effectiveness and acceptability of ALTs [8], and more specifically ALTs for older people [5], [9]. There has however not been a review covering the economic/cost analyses for the use of ALTs that could facilitate aging in place amongst older people, leaving an important gap in the research that this review specifically aims to address. A specific example of this gap is illustrated by the findings from a recent Scottish ‘smart technology’ study [10]. This report stated that the ALTs for older people used in their study could be accepted by users and staff. The ALTs were effective in reducing the use of certain health care resources; however the cost of the intervention was not considered so an economic analysis could not be performed [10]. Even if the effectiveness of ALTs for older people is proven to be universally accepted, as with any new health technology, it is important that the costs and benefits of widespread adoption are measured so decision makers in publically and privately funded healthcare systems can make evidence based decisions [11]. The lack of such research was evident in a recent overview [12] of systematic reviews that highlighted several important gaps in the telecare literature, one being economic analysis.

## Methods

### Search strategy

A search was undertaken in the two major health economic evaluation databases: NHS Economic Evaluation Database (NHS EED) and The Health Economic Evaluations Database (HEED) in July of 2012; details of the search strategy can be found in Text S1. NHS EED includes structured abstracts of quality-assessed full economic evaluations of health care technologies, with the only criterion for inclusion being that the study is a full economic evaluation. Like NHS EED, HEED includes full economic evaluations, and also includes cost analyses, cost-of-illness studies, and reviews of applied studies that include economic data. The search was systematic and reproducible. We used double reviewers for selected abstracts, with a third reviewer to settle any disagreements.

*Assisted living technologies for older people*, is a topic without a clearly defined taxonomy; therefore the extant literature is described by a range of keywords. Potential keywords were compiled from ‘on-topic’ papers and mesh terms found in scoping searches conducted between March and May 2012. A list of 91 potential keywords was compiled and each word or phrase was searched in NHS EED and HEED. For full keyword list, see Text S1.

### Selection criteria

Abstracts were reviewed to identify studies in English, comparing costs (cost-minimization studies) and cost and outcomes (full economic evaluations), published in peer reviewed journals. The ALTs were limited to those designed for older people (mean age in study 65+) that enable them to live independently in their residential home/age in place. No specific comparators were excluded.

Exclusion criteria were:

- Studies aimed at younger populations (average age under 65 or ages not reported).
- Studies not of human participants (theory or modelling papers).
- Studies based outside of the individuals' own homes.
- Interventions involving trained in-home professionals.
- Interventions involving home medical treatment, diagnosis and management were excluded, as were telecare interventions that ‘only *facilitates the exchange of data between patients and care professionals*’ [5] unless they specifically seek to enable independence.
- Studies not including comparisons of costs and/or outcomes to qualify as economic evaluations.

### Quality assessment

Studies were assessed using the methods recommended by the Campbell and Cochrane Economic Methods Group [13]. These include the methods outlined in the Cochrane handbook and more recent developments [14] [15], including methods to incorporate an economic perspective into the GRADE evidence system [16]. Current guidance from the CCEMG on quality assessment of cost-analyses is to use relevant components from one of two checklists: Jefferson et al. (1995) and Drummond et al. (2005) [17] [11]. In this study we opted to use that provided by Drummond et al. (2005).

### Data synthesis

Our intention was to apply specialist quantitative methods, including meta-analysis, to our results but, due to the poor and varied methodological quality of the included studies, we were not able to do so; therefore we employed a narrative synthesis approach. With the heterogeneity of the included studies the straightforward juxtaposition method of narrative synthesis, as recommended by the Cochrane Handbook [18], is the most appropriate. Therefore, the aim of this review was to find evidence for, or against, the cost-effectiveness of ALTs for older people to age in place as synthesized narratively across both cost and economic analysis studies; and to consider what the key determinants were of these outcomes across settings, rather than provide some pooled estimate that might not be applicable to any setting.

### Data Extraction Strategy

For the systematic review, the following six categories of data (listed below) were selected for extraction. Definition-classification data were also extracted, due to the fact that the assisted living technology field is lacking a standardized taxonomy of the technologies being assessed, as discussed by [8]; those results are presented in Table S1. For detailed subcategories of the data extraction strategy see Text S2.

Categories of data

1. Number of studies identified
2. Study identification and key elements
3. Source of cost data
4. Study perspective
5. Main outcomes
6. Data analysis: Critical Assessment of Economic Evaluation checklist (Table S2)

## Search Results

### 1. Number of studies identified

A total of 1,955 abstracts were identified and were screened for inclusion. From these studies, 34 were selected for further assessment and the full papers obtained and assessed. From there eight papers met the inclusion criteria for the review (Figure S1).

### 2. Study identification and key elements

Seven studies were conducted in the USA, with costs reported in US Dollars [19]–[25]. The remaining study [26] was conducted in Canada and reported in Canadian Dollars. The settings varied between remote rural, rural, and urban locations. The price year is important as it allows researchers to calculate how applicable the results could be to current practice. It was only explicitly stated in one study [22] and not stated in the others; where, at best, the price year could be estimated by publication/study date though that is not recommended practice [11].

Five studies were formal economic evaluations [19]–[23] comparing costs and consequences of two or more interventions [11]. Three were cost-minimisation analyses, which only compared costs as the interventions were assumed to have equal effect [24]–[26]. None of these three studies justified this assumption and the current consensus within the economic evaluation literature is that the adoption of a cost-minimisation framework is rarely justified [27].

From the eight studies, one considered only *Home and Environmental Modifications*, [19]; six considered *Telemedicine* [20]– and one study considered both [24]. The taxonomy and types of ALTs varied across studies see Table S1 for a detailed description of the ALTs and associated definitions.

### 3. Source of Cost Data

The cost data on the resource use was derived from National Patient Care Database at AAC, or Veterans Association records in 4 studies [6], [8], [10], [13], and was primarily costs rather than charges to a third party payer. Hospital level data was used to estimate costs in one study [7] and acquisition costs for the technology (with these costs based upon the retail price where equipment was donated) in one [9]. The source of cost data was not stated in the remaining two studies [11], [12]. The consequence of this is that it is not possible to translate the findings of these studies into the current time period.

### 4. Study perspective

From the eight papers, two did not specify the perspective of the study [19], [20]. One took the perspective of a public health system [26]. Two papers took the perspective of a hospital/health care provider [21], [22]. Two took the perspective of the Veterans Administration in the US [24], [25] and one took the perspective of the direct provider/purchaser [23].

### 5. Main Outcomes

#### Study Design

Of the eight identified studies, five were conducted as part of randomised control trials [19], [21]–[23], [25], two were conducted as part of quasi-experimental studies [20], [26]. Of these two, one did not state the design beyond having control and intervention groups [20] and the other had no control group because it stated that having one would be unethical [26]. The eighth study was a retrospective matched comparative study [24].

#### Costs studied

All of the studies reported on costs to the payer of providing the intervention. There was little reporting of the included costs consequent to the interventions. For example, downstream costs such as any changes in hospital and emergency care admission as a result of the intervention.

Changes consequent to the intervention were reported by Mann et al. (1999) as care aide costs, nurses, case managers, occupational and physical therapists and speech pathologists, nursing home stays and hospital costs [19]. Johnston et al. (2000) included costs of pharmacy services, laboratory, physician visits, Emergency Department (ED) visits, and inpatient treatment as well as the direct costs of home healthcare [20]. Noel & Vogel (2000) accounted for home visits, hospitalisations and ED visits [21]. Costs for clinic visits, hospitalisation, and transport were reported by Noel & Vogel (2004) [22]. Vincent et al. (2006) included hospital stays and home care services [26]. Finkelstein et al. (2006) inferred that there was a reduction in downstream direct cost evidenced by the documentation of fewer discharges to higher levels of care within the two intervention groups [23]. Bendixen et al. (2009) only reported future health care costs [24] and Wray et al. (2010) included outpatient, inpatient, and nursing home need [25]. None of the studies reported on indirect costs i.e. productivity losses borne by society due to health issues.

#### Patient characteristics

All studies had a mean patient and/or carer population age of above 65 in both intervention and control groups. Of the included studies, the overall mean population age was 72 years. Ages ranged from an intervention group mean of 68 years [21] to a mean of 81 years [26].

The split between male and female study participants varied between studies. Three studies had a higher percentage of women than men with 70% women [19], 59% [20], and 71% [26]. Two studies had a higher percentage of men with 95% [21], and 97% [22]. One study had an approximately even number of men and women [23] and two studies did not state the difference, both of which were working with US armed forces veterans [24], [25].

Four studies had a patient population comprised of newly diagnosed patients with a wide range of complex co-morbidities [20] [21] [22] [23]. One study considered interventions for home-based frail older people [19]. Another study looked at a patient population of veterans living at home with complex co-morbidities [24] and dementia [25]. In a further two studies, the focus was on ‘live-in’ caregivers, with one focusing on a close relative (as the caregiver) of veterans living at home with moderate to severe dementia [25] and the other targeting a close relative (as the caregiver) who lived with an older person who has a co-morbidity/barrier to independent living [26].

#### Primary outcomes of studies

Four of the studies reported effects on one or more outcomes [19], [20], [22], [24]. Mann (1999) reported on the effectiveness of assistive technologies and home environmental interventions using a variety of measures of functional independence as well as pain [19]. Johnston et al. (2000) reported on costs in a cost-effectiveness study of supportive care [20]. Noel et al (2004) used three quality indicators as outcomes (medication compliance, knowledge of disease, and ability for self-care), as well as reporting the degree of patient satisfaction [22]. Lastly, Bendixen et al. (2009) measured serum glucose, cognitive status, functional level, patient satisfaction with care and self-rated health status and quality of life [24]. Although used in a non comparative way, Vincent et al. (2006) used the SF-12 [28] to capture quality of life in the cohort in their cost analysis (as well as a measure of care giver burden) allowing for the possibly of maximizing gain across different technologies and sectors (allocative efficiency) [26]; the rest of the studies reported intervention-specific measures, with the aim of achieving increases in specific outcomes using the fewest resources (technical efficiency).

### 6. Data analysis: Critical Assessment of Economic Evaluation checklist

To determine if the evaluations were of acceptable quality, they were all assessed according to a well-recognized economic evaluation checklist [11]. The checklist (Table S2) was adapted from the Drummond et al. (2005) checklist and was used to illustrate the assessment of the validity of results found in this review. It is not expected that every economic evaluation satisfy all checklist criteria, though the systematic application of these questions can assist in the identification of strengths and weaknesses amongst the papers in this review. Table S2 illustrates the result of the systematic critical assessment of the data, demonstrating which studies present a sound economic evaluation. According to the assessment methods recommended by the Campbell and Cochrane Economic Methods Group, all the studies were judged to have low methodological quality [11].

The methodological quality of the studies is a reflection of the heterogeneous data on costs and outcomes reported across studies. Nevertheless, five studies reported that the intervention had lower costs (at least in the short-term) than the comparator group [19], [21]–[23], [25]. One study, where measures were taken before and after the introduction of telesurveillance, reported the intervention to have lowered healthcare expenditure in the intervention group, though there was no control group due to ethical reasons [26]. Another study found that there was no difference in costs between the intervention group and comparator, although after the intervention there were increases in clinic visits but decreases in hospital and nursing home stays for the intervention group [24]. One study found that the total mean costs of care were lower than in the control once the costs of home-healthcare were excluded [20].

Three studies reported non-monetary benefits [19], [20], [22]. The first found functional independence and mobility declined for both intervention and control group, but considerably more in the control; and that pain increased significantly less for the intervention than the control but physical independence, mobility occupation, and social integration were unchanged [19]. The second reported an association with improved medication compliance, knowledge of disease, and increased patient satisfaction [20]. The third found no significant effects on cognitive status, functional level, satisfaction with care or self-rated health status [22]. One study measured functional decline in terms of emergency and non-emergency care and found ALTs associated with reductions in hospital and nursing home stays [24]. No study included a preference based outcome measure meaning that there was no consideration of the value users of the ALTs place on the reported outcomes.

## Discussion

There is a potential for growth in the market for ALTs, although the growth of this market may not have developed as quickly as expected [29]. One reason for this may be the lack of studies proving the economic viability of ALTs [30]–[32]. Clearly there is a need for further economic evaluation of the ALTs that enable aging in place for older people [12]. This systematic review highlights a number of important issues. Firstly, more high quality studies investigating the cost-effectiveness of ALTs are required The results of the *Whole System Demonstrator* programme in the United Kingdom may help in this process, although there is still a lack of rigorous cost-effective work [5]. Cost-effectiveness studies can be an important source of information for public and private providers and more studies could help to stimulate the ALT market. A possible reason why there have been few cost-effectiveness studies of ALTs may be because new technology is consistently developing and as technologies ‘age’ the costs can change.

Secondly, an increased focus on defining and measuring the benefits of ALTs is required. Outcome measurement is not well-developed in social care and the aging process. There is important research on-going but it has yet to make it into the wider evaluation of ALT literature. The included studies lacked a consistent use of a health related quality of life measures as an outcome; using such measures would allow issues concerning allocative efficiency to be considered, whereas the outcomes measured in this review were better placed to answer questions about technical efficiency. Focusing solely on monetary change as an outcome is problematic as it is possible for an intervention to have an ‘economic benefit’ but the health and wellbeing of participants may not necessarily improve.

Furthermore, health related quality of life should not be limited to just physical aspects of well-being but extended to the mental wellbeing of participants as well. With ALTs allowing older people to age in place, the change in mental outlook should be measured as an outcome in future economic evaluations. The capabilities approach would seem to be an exciting opportunity, as it focuses on self-reported wellbeing in older people as defined in a broader sense instead of exclusively on health [33]. The ICECAP-O (ICEpop CAPability measure for Older people) is one example of this approach as it contains subjective measures including a dimension solely about independence [34]. This instrument has recently been used as the measure for a secondary outcome in a UK based study involving older people, though the tool is still new and research regarding its validation is ongoing [35]. Utilizing a preference-based instrument provides decision makers with additional information that includes valuations of the states the tools measure. Two studies in this review [24], [26] actually collected data that could have been used to perform an economic evaluation and so demonstrate whether the interventions were cost effective but no such results were presented. Measures like the ICECAP-O may be able to better capture changes in the overall wellbeing of older people using ALTs to age in place specifically, rather than solely focusing on function or health related quality of life (e.g. EQ-5D) [36]. Such tools are promising and although research is on-going, more is needed [36].

Thirdly, there is a need to consider where benefits accrue, and so which perspective should be taken. Many ALTs produce benefits that accrue to individuals, or groups, involved in the care needs of the user, such as: household members, wider family and carers, as well as the state, in the form of local and national funders. This presents a challenge for research and suggests that the best approach to evaluation may be cost-benefit analysis, where wider notion of costs and benefits can be valued.

Finally, there is a need for a clear taxonomy describing ALTs. ALTs encompass a broad range of devices: from basic home adaptations to help frail older people obtain control over their environment, to advances in telecare and the cutting edge technology found in ‘‘smart homes’. With such diversity, there is uncertainty within the literature as to the categorization and terminology of technologies [7]. Studies and reviews have called for more research and work on these issues [8]. We concur that more clarity regarding taxonomy and the outcomes of ALTs is required. Additionally, research on ALTs specifically facilitating aging in place for older people could benefit from developments in taxonomy so the issues can be analysed separately from telecare in the management of chronic conditions.

## Conclusion

The strengths of our study are that the search was systematic and reproducible. The identified studies were quality assessed according to the criteria proposed by the Campbell and Cochrane Economic Methods Group [17]; which deemed the methodological quality to be weak across studies. The inclusion criterion was strict as we wanted to target only ALTs that assist with aging in place opposed to telecare in the management of chronic conditions; though as a result there may be related studies (i.e. modelling papers) that were excluded. The search was limited to full text articles available in the English language, though both HEED and NHS EED do not limit their searches to the English language and provide the structured abstracts are written in English. All of the studies were based in North American settings which may not be universally applicable to other international settings. Two of the studies had the United States Department of Veterans Affairs as its perspective which is publicly funded though neither study took into account the cost of the intervention in its analysis.

The review has highlighted some key areas of research for ALTs, social care and aging in place. ALTs may be an innovative solution to the problems posed by aging populations, but more research concerning their cost-effectiveness is required.

## Supporting Information

Figure S1 **Review and Selection of Articles.** This figure shows the stages of the systematic review selection process as detailed with the number of studies progressing through the inclusion and exclusion criteria.
(DOCX)

Table S1 **ALT Definition Classification.** This table displays the results of the varying taxonomy across the included studies as categorized by how each ALT was titled and the specific details of what the technology was in each study. The two main categories are ‘Home Modifications (Table A)’ and ‘Remote Patient-Health Professional Communication’ (Table B).
(DOCX)

Table S2 **Critical Assessment of Economic Evaluation checklist.** This checklist was adapted from the Drummond et al. (2005) checklist and was used to illustrate the assessment of the validity of results found in this review, demonstrating which studies present a sound economic evaluation.
(DOCX)

Checklist S1 **PRISMA 2009 Checklist.** PRISMA stands for Preferred Reporting Items for Systematic Reviews and Meta-Analyses and is an evidence-based minimum set of items for reporting in systematic reviews and meta-analyses. Checklist S1 shows how the authors have completed the 27-item PRISMA 2009 checklist to accompany the PRISMA 2009 flow diagram (Flow Diagram S1).
(DOCX)

Flow Diagram S1 **PRISMA 2009 Flow Diagram.** PRISMA stands for Preferred Reporting Items for Systematic Reviews and Meta-Analyses and is an evidence-based minimum set of items for reporting in systematic reviews and meta-analyses. Flow Diagram S1 is the PRISMA 2009 four-phase flow diagram of the systematic review process to accompany the PRISMA 2009 Checklist (Checklist S1).
(DOCX)

Text S1 **Keywords & Search Strings used for Systematic Search.** This document lists the keywords and database search strategy used for the systematic search.
(DOCX)

Text S2 **Data Extraction Strategy.** This document presents the full outline of the data extracted from the included studies.
(DOCX)

## References

[1] Gitlin LN (2003) Conducting Research on Home Environments: Lessons Learned and New Directions. The Gerontologist 43: 628–637. 14570959 10.1093/geront/43.5.628

[2] Wiles JL, Leibing A, Guberman N, Reeve J, Allen RES (2011) The Meaning of “Ageing in Place” to Older People. The Gerontologist.

[3] Beer JM, Smarr C, Chen TL, Prakash A, Mitzner TL, et al. (2012) The domesticated robot: Design guidelines for assisting older adults to age in place; 2012 5–8 March 2012. pp. 335–342. 10.1145/2157689.2157806 PMC6592031 31240279

[4] World Health Organization (2014) Definition of an older or elderly person. Proposed Working Definition of an Older Person in Africa for the MDS Project.

[5] Steventon A, Bardsley M, Billings J, Dixon J, Doll H, et al (2012) Effect of telehealth on use of secondary care and mortality: findings from the Whole System Demonstrator cluster randomised trial. British Medical Journal 344.. 10.1136/bmj.e3874 PMC3381047 22723612

[6] Sood S, Mbarika V, Jugoo S, Dookhy R, Doarn CR, et al (2007) What is telemedicine? A collection of 104 peer-reviewed perspectives and theoretical underpinnings. Telemedicine Journal and E-Health 13: 573–590. 17999619 10.1089/tmj.2006.0073

[7] Halford S, Lotherington AT, Obstfelder A, Dyb K (2010) GETTING THE WHOLE PICTURE? New information and communication technologies in healthcare work and organization. Information Communication & Society 13: 442–465.

[8] Martin S, Kelly G, Kernohan G, McCreight B, Nugent C (2008) Smart home technologies for health and social care support. Cochrane Database of Systematic Reviews 2008. The Cochrane Library: John Wiley & Sons. 10.1002/14651858.CD006412.pub2 PMC11186705 18843715

[9] Barlow J, Singh D, Bayer S, Curry R (2007) A systematic review of the benefits of home telecare for frail elderly people and those with long-term conditions. Journal of Telemedicine and Telecare 13: 172–179. 17565772 10.1258/135763307780908058

[10] Bowes A, McColgan G (2006) Smart technology and community care for older people: innovation in West Lothian, Scotland. Age Concern Scotland.

[11] Drummond MF, Stoddart GL, Torrance GW (2005) Methods for the Economic Evaluation of Health Care Programmes; Publications OM, editor.

[12] Bahaadinbeigy K, Yogesan K, Wootton R (2010) Gaps in the systematic reviews of the telemedicine field. Journal of Telemedicine and Telecare 16: 414–416. 20841383 10.1258/jtt.2010.100505

[13] Shemilt I, Byford S, Drummond M, Eisenstein E, Knapp M, et al. (2008) Incorporating Economics Evidence. In Higgins J, Green S (eds) Cochrane Handbook for Systematic Reviews of Interventions. Chichester: Hohn Wiley & Sons.

[14] Shemilt I, McDaid D, Marsh K, Henderson C, Bertranou E, et al (2013) Issues in the incorporation of economic perspectives and evidence into Cochrane reviews. Systematic reviews 2.. 10.1186/2046-4053-2-83 PMC3849717 24050504

[15] Shemilt I, Mugford M, Donaldson C, Vale L, Marsh K (2010) Evidence-based decisions and economics: lessons for practice. Evidence based economics. London: Wiley Books.

[16] Brunetti M, Shemilt I, Pregno S, Vale L, Oxman AD, et al (2013) GRADE guidelines: 10. Considering resource use and rating the quality of economic evidence. Journal of Clinical Epidemiology 66: 140–150. 22863410 10.1016/j.jclinepi.2012.04.012

[17] Jefferson T, Demicheli V, Entwistle V (1995) ASSESSING QUALITY OF ECONOMIC SUBMISSIONS TO THE BMJ. British Medical Journal 311: 393–394. 10.1136/bmj.311.7001.393c PMC2550462 7640570

[18] Higgins JPT, (editors) GS (2009) Cochrane Handbook for Systematic Reviews of Interventions Version 5.0.2 [updated September 2009]. The Cochrane Collaboration.

[19] Mann WC, Ottenbacher KJ, Fraas L, Tomita M, Granger CV (1999) Effectiveness of assistive technology and environmental interventions in maintaining independence and reducing home care costs for the frail elderly - A randomized controlled trial. Archives of Family Medicine 8: 210–217. 10333815 10.1001/archfami.8.3.210

[20] Johnston B, Wheeler L, Deuser J, Sousa KH (2000) Outcomes of the Kaiser Permanente tele-home health research project. Archives of Family Medicine 9: 40–45. 10664641 10.1001/archfami.9.1.40

[21] Noel HC, Vogel DC (2000) Resource costs and quality of life outcomes for homebound elderly using telemedicine integrated with nurse case management. Care Management 6(5): : 22–24, 26–82, 30–31.

[22] Noel HC, Vogel DC, Erdos JJ, Cornwall D, Levin F (2004) Home telehealth reduces Healthcare costs. Telemedicine Journal and E-Health 10: 170–183. 15319047 10.1089/tmj.2004.10.170

[23] Finkelstein SM, Speedie SM, Potthoff S (2006) Home telehealth improves clinical outcomes at lower cost for home healthcare. Telemedicine Journal and E-Health 12: 128–136. 16620167 10.1089/tmj.2006.12.128

[24] Bendixen RM, Levy CE, Olive ES, Kobb RF, Mann WC (2009) Cost Effectiveness of a Telerehabilitation Program to Support Chronically Ill and Disabled Elders in Their Homes. Telemedicine Journal and E-Health 15: 31–38. 19199845 10.1089/tmj.2008.0046

[25] Wray LO, Shulan MD, Toseland RW, Freeman KE, Vasquez BE, et al (2010) The Effect of Telephone Support Groups on Costs of Care for Veterans With Dementia. Gerontologist 50: 623–631. 20507926 10.1093/geront/gnq040

[26] Vincent C, Reinharz D, Deaudelin I, Garceau M, Talbot LR (2006) Public telesurveillance service for frail elderly living at home, outcomes and cost evolution: a quasi experimental design with two follow-ups. Health and Quality of Life Outcomes 4.. 10.1186/1477-7525-4-41 PMC1562360 16827929

[27] Briggs AH, O'Brien BJ (2001) The death of cost-minimization analysis? Health Economics 10(2): 179–184. 11252048 10.1002/hec.584

[28] Burdine JN, Felix MRJ, Abel AL, Wiltraut CJ, Musselman YJ (2000) The SF-12 as a population health measure: An exploratory examination of potential for application. Health Services Research 35: 885–904. 11055454 PMC1089158

[29] Lushai G, Cox T (2012) Assisted Living Technology, a market and technology review. University of the West of England.

[30] Aanesen M, Lotherington AT, Olsen F (2011) Smarter elder care? A cost-effectiveness analysis of implementing technology in elder care. Health Informatics Journal 17: 161–172. 21937460 10.1177/1460458211409716

[31] Vimarlund V, Olve N-G (2005) Economic analyses for ICT in elderly healthcare: questions and challenges. Health Informatics Journal 11: 309–321.

[32] Whitten PS, Mair FS, Haycox A, May CR, Williams TL, et al (2002) Systematic review of cost effectiveness studies of telemedicine interventions. BMJ 324: 1434–1437. 12065269 10.1136/bmj.324.7351.1434 PMC115857

[33] Grewal I, Lewis J, Flynn T, Brown J, Bond J, et al (2006) Developing attributes for a generic quality of life measure for older people: Preferences or capabilities? Social Science & Medicine 62: 1891–1901. 16168542 10.1016/j.socscimed.2005.08.023

[34] Coast J, Flynn TN, Natarajan L, Sproston K, Lewis J, et al (2008) Valuing the ICECAP capability index for older people. Social Science & Medicine 67: 874–882. 18572295 10.1016/j.socscimed.2008.05.015

[35] Henderson C, Knapp M, Fernández J, Beecham J, Hirani SP, et al (2013) Cost effectiveness of telehealth for patients with long term conditions (Whole Systems Demonstrator telehealth questionnaire study): nested economic evaluation in a pragmatic, cluster randomised controlled trial. BMJ 346.. 10.1136/bmj.f1035 23520339

[36] Davis JC, Bryan S, McLeod R, Rogers J, Khan K, et al (2012) Exploration of the association between quality of life, assessed by the EQ-5D and ICECAP-O, and falls risk, cognitive function and daily function, in older adults with mobility impairments. Bmc Geriatrics 12.. 10.1186/1471-2318-12-65 PMC3534357 23095570

