# Supplementary material for: Can Aging in Place Be Cost Effective? A Systematic Review
Source: PLoS One. 2014 Jul 24;9(7):e102705. doi: 10.1371/journal.pone.0102705 (PMC4109953; doi:10.1371/journal.pone.0102705)
Supplement: Figure S1 — Review and Selection of Articles. This figure shows the stages of the systematic review selection process as detailed with the number of studies progressing through the inclusion and exclusion criteria. (DOCX) [file pone.0102705.s001.docx]

**Figure S1- Review and Selection of Articles.**

1,955 Potentially relevant citations from electronic

database search HEED (892) and NHS EED (1,063)

1,921 Citations excluded based on review of abstract

34 Potentially relevant articles identified for further review

26 Articles excluded based on review of article

5 Intervention was not designed primarily as an ALT

4 Intervention primarily used human resource

3 Criteria for intervention setting not met

5 Criteria for population not met

3 Criteria for type of study not met

1 Full text was not in English

5 Duplicates

8 Articles included in systematic review

­­­­­­
