# Supplementary material for: Can Aging in Place Be Cost Effective? A Systematic Review
Source: PLoS One. 2014 Jul 24;9(7):e102705. doi: 10.1371/journal.pone.0102705 (PMC4109953; doi:10.1371/journal.pone.0102705)
Supplement: Table S1 — ALT Definition Classification. This table displays the results of the varying taxonomy across the included studies as categorized by how each ALT was titled and the specific details of what the technology was in each study. The two main categories are ‘Home Modifications (Table A)’ and ‘Remote Patient-Health Professional Communication’ (Table B). (DOCX) [file pone.0102705.s002.docx]

**Table S1-ALT Definition Classification.**

Due to the fact that the assisted living technology field is lacking a standardized taxonomy of the technologies being assessed, definition-classification data were extracted as illustrated in the tables below. The two main categories are those ALTs which can be considered: *Home Modifications* (Table A) or *Remote Patient-Health Professional Communication* (Table B).

**Table A) Home Modifications**

| **Year, Authors** | **Country** | **Name(s) of ALT in study** | **Details of ALT** |
| --- | --- | --- | --- |
| 1999, Mann W C, Ottenbacher K J, Fraas L, Tomita M, Granger C V | USA | 1. Assistive technology | Canes, walkers, and bath benches added to home. |
|  |  | 2. Home environment interventions | Ramps, lowering of cabinets, removal of throw rugs. |
| 2009, Bendixen R M, Levy C E, Olive E S, Kobb R F, Mann W C | USA | Telerehabilitation^1^ | A program including adaptive equipment and environmental modifications focused on self-care and safety within the home. |
| ^1^This intervention called ‘Telerahabilitation’ used a two phase approach including both a *home modification* phase listed in Table 2a and a *Remote patient-health professional communication* phase listed in Table 2b. | | | |

**Table B) Remote Patient-Health Professional Communication**

| **Year, Authors** | **Country** | **Name(s) of ALT in study** | **Details of ALT** |
| --- | --- | --- | --- |
| 2000, Johnston B, Wheeler L, Deuser J, Sousa K H | USA | Tele-Home Health | Video equipment installed in the homes of older people which allowed for:  24 hour access to Home Health Nurses through the Home Health Department by using remote video visit technology during normal working hours and on-call hours.  The video system included peripheral equipment for assessing cardiopulmonary status. In depth triage could be provided without older people having to leave the home. |
| 2000, Noel H C, Vogel D C | USA | Telemedicine | Home surveillance technology to monitor the condition of the older person by transmitting routine physiological data to a nurse-staffed, central nurse station.  The hospital unit accommodated peripherals for blood pressure, pulse rate, pulse oxymetry, camera, temperature, lung sounds, and electrocardiogram.  A voice system reminded the participants to take their medication and to use peripherals during specific timeframes. |
| 2004 Noel H C, Vogel D C, Erdos J J, Cornwall D, Levin F | USA | 1. Home telehealth  2. Home telemedicine | Older people received units that used standard phone lines to communicate with the hospital. FDA-approved peripheral devices monitored vital signs. Out of range data triggered electronic alerts to nurse case managers.  No live video or audio was incorporated in either direction. |
| 2006 Vincent C, Reinharz D, Deaudelin I, Garceau M, Talbot L R | Canada | Telesurveillance Service | Technology consists of a telephone and a small battery-powered wireless emergency call transmitter with bi-directional communication capabilities (speaker-receiver) between the older person and the (nurse staffed) calling center.  The wireless unit can be worn as a medallion or bracelet, is impact and water-resistant, and can be used to place emergency calls, or to answer the phone when the user is not near the base set.  The telephone is equipped with oversized, illuminated buttons and a light ergonomic handset, which is compatible with hearing aids.  This model possesses many other special features including bi-directional communication up to 30 meters from the phone, and voice reminders (e.g., for medication, catheters, glycaemia, special diet, prescribed exercises, medical appointments, important social functions, daily activity tasks) that can be set for specific times (daily, weekly or only once). Six reminders can be stored simultaneously and programmed remotely.  The older person can also answer and speak on the telephone remotely simply by pressing the emergency button, without picking up the phone.  Another available function is the ability to get the time, day and date by pressing a button. |
| 2006 Finkelstein S M, Speedie S M, Potthoff S | USA | 1. TeleHomeCare | A program integrating information, telecommunications, and physiological monitoring technology for patients at home in lieu of visits to the home by healthcare workers. |
|  |  | 2. Home telehealth | Virtual video visits, and virtual video visits with physiological monitoring.  The technology set up in the older people’s homes consisted of a set-top box connected to the subject’s television set and telephone line. A lightweight, variable focus eyeball camera was placed on the box with a 6-foot tether for easy positioning so that the camera, and not the subject, could be moved to transmit real-time pictures of wounds, swollen ankles, etc. An automatic focusing adjustment and freeze-frame video mode increase chances of good quality images being transmitted for evaluation.  Subjects in the monitoring group also received physiologic monitoring devices appropriate for their underlying health condition. |
|  |  | 3. Virtual Video Visits | Virtual visits between a skilled home healthcare nurse and chronically ill patients at home.  The nurses initiated virtual visits by telephoning the subject (using a set-top box with built in camera used at each Home Health Care agency); the subject answered the call to proceed with the virtual visit. The nurse controlled audio and video adjustments at both sites. The nurse entered all standard medical charting data for TeleHomeCare into a clinical information system. |
| 2009 Bendixen R M, Levy C E, Olive E S, Kobb R F, Mann W C | USA | Telerahabilitation^1^ | Care coordinators remotely monitored their patient’s vital signs and provided education and self-management strategies for decreasing the effects of chronic illnesses and functional decline. |
| ^1^This intervention called ‘Telerahabilitation’ used a two phase approach including both a *home modification* phase listed in Table 2a and a *Remote patient-health professional communication* phase listed in Table 2b. | | | |
