# Supplementary material for: Can Aging in Place Be Cost Effective? A Systematic Review
Source: PLoS One. 2014 Jul 24;9(7):e102705. doi: 10.1371/journal.pone.0102705 (PMC4109953; doi:10.1371/journal.pone.0102705)
Supplement: Table S2 — Critical Assessment of Economic Evaluation checklist. This checklist was adapted from the Drummond et al. (2005) checklist and was used to illustrate the assessment of the validity of results found in this review, demonstrating which studies present a sound economic evaluation. (DOCX) [file pone.0102705.s003.docx]

| **Answer** | **Checklist Questions and answers** |
| --- | --- |
| **1. Was a well-defined question posed in answerable form?** | |
| YES | Mann et al. 1999; Johnston et al., 2000; Noel and Vogel, 2000; Noel et al., 2004; Vincent et al., 2006; Finkelstein et al., 2006; Bendixen et al., 2009; Wray et al., 2010 |
| NO | None |
| **2. Was a comprehensive description of the competing alternatives given?** | |
| YES | Mann et al. 1999; Johnston et al., 2000; Noel and Vogel, 2000; Noel et al., 2004; Finkelstein et al., 2006; Bendixen et al., 2009; Wray et al., 2010 |
| NO | None |
| *N/A* | No alternative considered Vincent et al., 2006 |
| **3. Were all the important and relevant costs for each alternative identified?** | |
| YES | Johnston et al., 2000; Finkelstein et al., 2006 |
| NO | Intervention costs were not reported Bendixen et al., 2009; Wray et al., 2010 |
| NO | Only intervention costs were collected and no cost savings associated with the outcome: Mann et al. 1999; Noel and Vogel, 2000; Noel et al., 2004 |
| *N/A* | No alternative considered: Vincent et al., 2006 |
| **4. Were costs measured accurately in appropriate physical units?** | |
| YES | Mann et al. 1999; Johnston et al., 2000; Noel et al., 2004; Finkelstein et al., 2006; Bendixen et al., 2009; Wray et al., 2010 |
| NO | Noel and Vogel, 2000; Vincent et al., 2006 |
| **5. Were costs valued credibly?** | |
| YES | Mann et al. 1999; Johnston et al., 2000; Noel et al., 2004; Vincent et al., 2006; Finkelstein et al., 2006; Bendixen et al., 2009; Wray et al., 2010 |
| NO | Noel and Vogel, 2000 |
| **6. Were costs adjusted for differential timing?** | |
| YES | None |
| NO | All: None of the studies were over two years so discounting was not needed |
| **7. Was an incremental analysis of costs of alternatives performed?** | |
| YES | None |
| NO | All: None of the studies performed analysis using an incremental cost-effectiveness ratio |
| **8. Was allowance made for uncertainty in the estimation of costs?** | |
| YES | None |
| NO | All: Sensitivity analysis was not performed |
| **9. Did the presentation and discussion of study results include all issues of concern to users?** | |
| YES | Mann et al. 1999; Johnston et al., 2000; Noel and Vogel, 2000; Noel et al., 2004; Finkelstein et al., 2006; Vincent et al., 2006 |
| NO | Intervention costs were not reported: Bendixen et al., 2009; Wray et al., 2010 |
| *N/A = category not applicable to identified study* | |

**Table S2-Critical Assessment of Economic Evaluation Checklist.**
