# Supplementary material for: Can Aging in Place Be Cost Effective? A Systematic Review
Source: PLoS One. 2014 Jul 24;9(7):e102705. doi: 10.1371/journal.pone.0102705 (PMC4109953; doi:10.1371/journal.pone.0102705)
Supplement: Checklist S1 — PRISMA 2009 Checklist. PRISMA stands for Preferred Reporting Items for Systematic Reviews and Meta-Analyses and is an evidence-based minimum set of items for reporting in systematic reviews and meta-analyses. Checklist S1 shows how the authors have completed the 27-item PRISMA 2009 checklist to accompany the PRISMA 2009 flow diagram (Flow Diagram S1). (DOCX) [file pone.0102705.s004.docx]

| **Section/topic** | **#** | **Checklist item** | **Reported on page #** |
| --- | --- | --- | --- |
| **TITLE** | | |  |
| Title | 1 | Identify the report as a systematic review, meta-analysis, or both. | Title page: page 1 |
| **ABSTRACT** | | |  |
| Structured summary | 2 | Provide a structured summary including, as applicable: background; objectives; data sources; study eligibility criteria, participants, and interventions; study appraisal and synthesis methods; results; limitations; conclusions and implications of key findings; ~~systematic review registration number~~. | Abstract: page 2 |
| **INTRODUCTION** | | |  |
| Rationale | 3 | Describe the rationale for the review in the context of what is already known. | Introduction: pages 3 & 4 |
| Objectives | 4 | Provide an explicit statement of questions being addressed with reference to participants, interventions, comparisons, outcomes, and study design (PICOS). | Selection criteria: page 5 & 6 |
| **METHODS** | | |  |
| Protocol and registration | 5 | Indicate if a review protocol exists, if and where it can be accessed (e.g., Web address) ~~if available, provide registration information including registration number.~~ | Available from authors upon request |
| Eligibility criteria | 6 | Specify study characteristics (e.g., PICOS, length of follow-up) and report characteristics (e.g., years considered, language, publication status) used as criteria for eligibility, giving rationale. | Methods, Selection criteria: page 5 |
| Information sources | 7 | Describe all information sources (e.g., databases with dates of coverage, contact with study authors to identify additional studies) in the search and date last searched. | Methods, search strategy: page 4 & 5 |
| Search | 8 | Present full electronic search strategy for at least one database, including any limits used, such that it could be repeated. | Methods, search strategy: page 4 & 5 and Text S1- Keywords & Search Stings used for systematic search |
| Study selection | 9 | State the process for selecting studies (i.e., screening, eligibility, included in systematic review ~~and, if applicable, included in the meta-analysis).~~ | Selection Criteria pages 5 & 6, Search results: page 7, and Figure S1 |
| Data collection process | 10 | Describe method of data extraction from reports (e.g., piloted forms, independently, in duplicate) and any processes for obtaining and confirming data from investigators. | Data synthesis: pages 6 & 7 and Text S2-Data extraction strategy |
| Data items | 11 | List and define all variables for which data were sought (e.g., PICOS, funding sources) and any assumptions and simplifications made. | Source of cost data: pages 8 & 9 and S2-Data extraction strategy |
| Risk of bias in individual studies | 12 | Describe methods used for assessing risk of bias of individual studies (including specification of whether this was done at the study or outcome level), and how this information is to be used in any data synthesis. | Drummond (1995) Checklist applied:  From page 6 of manuscript:  *Studies were assessed using the methods recommended by the Campbell and Cochrane Economic Methods Group. These include the methods outlined in the Cochrane handbook and more recent developments, including methods to incorporate an economic perspective into the GRADE evidence system. Current guidance from the CCEMG on quality assessment of cost-analyses is to use relevant components from one of two checklists: Jefferson et al. (1995) and Drummond et al. (2005). In this study we opted to use that provided by Drummond et al. (2005).(Table S2)* |
| Summary measures | 13 | State the principal summary measures (e.g., risk ratio, difference in means). | Main Outcomes: pages 9-11 |
| Synthesis of results | 14 | Describe the methods of handling data and combining results of studies, if done, including measures of consistency ~~(e.g., I~~^~~2~~^~~) for each meta-analysis.~~ | Narrative synthesis as described first on page 2 |

Page 1 of 2

| **Section/topic** | **#** | **Checklist item** | **Reported on page #** |
| --- | --- | --- | --- |
| Risk of bias across studies | 15 | Specify any assessment of risk of bias that may affect the cumulative evidence (e.g., publication bias, selective reporting within studies). | Drummond Checklist criteria, results illustrated in Table S2- Critical Assessment of Economic Evaluation checklist: page 20 |
| Additional analyses | 16 | Describe methods of additional analyses (e.g., sensitivity or subgroup analyses, meta-regression), if done, indicating which were pre-specified. | N/A |
| **RESULTS** | | |  |
| Study selection | 17 | Give numbers of studies screened, assessed for eligibility, and included in the review, with reasons for exclusions at each stage, ideally with a flow diagram. | PRISMA Flow diagram (Flow Diagram S1) and Figure S1 |
| Study characteristics | 18 | For each study, present characteristics for which data were extracted (e.g., study size, PICOS, follow-up period) and provide the citations. | Study identification and key elements: page 7 & 8 |
| Risk of bias within studies | 19 | Present data on risk of bias of each study and, if available, any outcome level assessment (see item 12). | N/A |
| Results of individual studies | 20 | For all outcomes considered (benefits or harms), present, for each study: (a) simple summary data for each intervention group (b) effect estimates and confidence intervals, ideally with a forest plot. | A) outcomes of studies: page 11 B) N/A |
| Synthesis of results | 21 | Present results of each meta-analysis done, including confidence intervals and measures of consistency. | N/A |
| Risk of bias across studies | 22 | Present results of any assessment of risk of bias across studies (see Item 15). | Table 1- Critical Assessment of Economic Evaluation checklist: page 20, Table S2 |
| Additional analysis | 23 | Give results of additional analyses, if done (e.g., sensitivity or subgroup analyses, meta-regression [see Item 16]). | N/A |
| **DISCUSSION** | | |  |
| Summary of evidence | 24 | Summarize the main findings including the strength of evidence for each main outcome; consider their relevance to key groups (e.g., healthcare providers, users, and policy makers). | Conclusion and Discussion: pages 13-16 |
| Limitations | 25 | Discuss limitations at study and outcome level (e.g., risk of bias), and at review-level (e.g., incomplete retrieval of identified research, reporting bias). | Conclusion and Discussion: pages 13-16 |
| Conclusions | 26 | Provide a general interpretation of the results in the context of other evidence, and implications for future research. | Conclusion and Discussion: pages 13-16 |
| **FUNDING** | | |  |
| Funding | 27 | Describe sources of funding for the systematic review and other support (e.g., supply of data); role of funders for the systematic review. | Page 21 |

*From:*  Moher D, Liberati A, Tetzlaff J, Altman DG, The PRISMA Group (2009). Preferred Reporting Items for Systematic Reviews and Meta-Analyses: The PRISMA Statement. PLoS Med 6(6): e1000097. doi:10.1371/journal.pmed1000097

For more information, visit: **www.prisma-statement.org**.

Page 2 of 2
