# Supplementary material for: Can Aging in Place Be Cost Effective? A Systematic Review
Source: PLoS One. 2014 Jul 24;9(7):e102705. doi: 10.1371/journal.pone.0102705 (PMC4109953; doi:10.1371/journal.pone.0102705)
Supplement: Flow Diagram S1 — PRISMA 2009 Flow Diagram. PRISMA stands for Preferred Reporting Items for Systematic Reviews and Meta-Analyses and is an evidence-based minimum set of items for reporting in systematic reviews and meta-analyses. Flow Diagram S1 is the PRISMA 2009 four-phase flow diagram of the systematic review process to accompany the PRISMA 2009 Checklist (Checklist S1). (DOCX) [file pone.0102705.s005.docx]

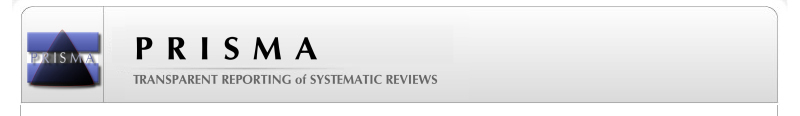
**PRISMA 2009 Flow Diagram**

Studies included in narrative synthesis
(n = 8)

Records identified through database searching
(n =1,955 )

Full-text articles excluded, with reasons
(n = 21)

5 Intervention was not designed primarily as an ALT

4 Intervention primarily used human resource

3 Criteria for intervention setting not met

5 Criteria for population not met

3 Criteria for type of study not met

1 Full text was not in English

Records excluded
(n =1,921)

Full-text articles assessed for eligibility
(n =29)

Records screened
(n =1,950)

Records after duplicates removed
(n =1,950)

## Identification

## Eligibility

## Included

## Screening
