# Supplementary material for: Can Aging in Place Be Cost Effective? A Systematic Review
Source: PLoS One. 2014 Jul 24;9(7):e102705. doi: 10.1371/journal.pone.0102705 (PMC4109953; doi:10.1371/journal.pone.0102705)
Supplement: Text S1 — Keywords & Search Strings used for Systematic Search. This document lists the keywords and database search strategy used for the systematic search. (DOCX) [file pone.0102705.s006.docx]

**Text S1-Keywords & Search Strings used for Systematic Search.**

The below keywords were entered as they appear into both the NHS Economic Evaluation Database (NHS EED) and the Health Economic Evaluation Database (HEED). The search option selected for the NHS EED keyword search was ‘Any Field’. The ‘Quick Search’ option for HEED was used with ‘All Data’ being the search option selected.

Automatic home

Aal

Activities of Daily Living

Aging in place

Aging in-place

Ambient assisted living

Ambient environment

Ambient object

Ambulatory health monitor

Assistive device

Assistive technology

AT

Assistive wheelchair

Automatic health monitor

Automatic house

Automatic sensor

Automatic services

Automatic wheelchair

Aware Home

Computer trends

E-health

Environment Design

Environment(al) control system(s)

Functional Independence Measure (FIM)

Health Services for the Aged

Health Services for the Aged/trends

Health telematic

Health-enabling technologies

Home Care Services

Home environmental interventions (EIs)

Home health monitor

Home telecare technologies

Implantable device

Information and communication technology (ICT)

Instrumental Activities of Daily Living to assess disability

Intelligent environment

Intelligent object

Interactive

Internet

Medical informatics

Medical telematic

Mobility

Personal health monitor

Phone

Power wheelchair

Remote control

Remote physiological monitoring

Robotic assistants

Self health monitor

Self-Help Devices/trends

Smart home

Smart home technology

Smart house

Smart house technology

Smart sensor

Smart service

Smart Wearable Garment technologies

Smart wheelchair

SMS

Social alarm

Social alert

Technology Initiative for Disabled and Elderly People (TIDE)

Tele-assistance

TELECARDIOLOGY

Telecare

Tele-care

Telecommunication

Teleconsultation

Tele-consultation

TELEDERMATOLOGY

Telehealth

Tele-health

Telehomecare

Tele-homecare

Telemanagement

Tele-management

Telematic

Telemedicine

Tele-medicine

Telemedicine/trends

Tele-monitor

Telemonitor

TELEPATHOLOGY

Telepharmacy

Tele-pharmacy

Telephone

Tele-rehabilitation

Video visits

Wearable device

Wearable sensor

Web
