# Supplementary material for: Can Aging in Place Be Cost Effective? A Systematic Review
Source: PLoS One. 2014 Jul 24;9(7):e102705. doi: 10.1371/journal.pone.0102705 (PMC4109953; doi:10.1371/journal.pone.0102705)
Supplement: Text S2 — Data Extraction Strategy. This document presents the full outline of the data extracted from the included studies. (DOCX) [file pone.0102705.s007.docx]

**Text S2-Data Extraction Strategy.**

**1. Number of studies identified**

(a) Included.

(b) Excluded (reasons for exclusion).

**2. Study identification and key elements**

(a) Author and year.

(b) Country of origin and currency reported.

(c) Type of economic evaluation.

(d) Type of intervention studied.

**3. Sources of cost data**

**4. Study perspective**

**5. Main outcomes**

(a) Design summary.

(b) Costs.

(c) Patient characteristics (sick, carers, veterans).

(d) Primary non-monetary outcome (if appropriate).

**6. Data analysis: Critical Assessment of Economic Evaluation checklist**

(1) Was a well-defined question posed in answerable form?

(2) Was a comprehensive description of the competing alternatives given?

(3) Were all the important and relevant costs for each alternative identified?

(4) Were costs measured accurately in appropriate physical units?

(5) Were costs valued credibly?

(6) Were costs adjusted for differential timing?

(7) Was an incremental analysis of costs of alternatives performed?

(8) Was allowance made for uncertainty in the estimates of costs?

(9) Did the presentation and discussion of study results include all issues of concern to users?
